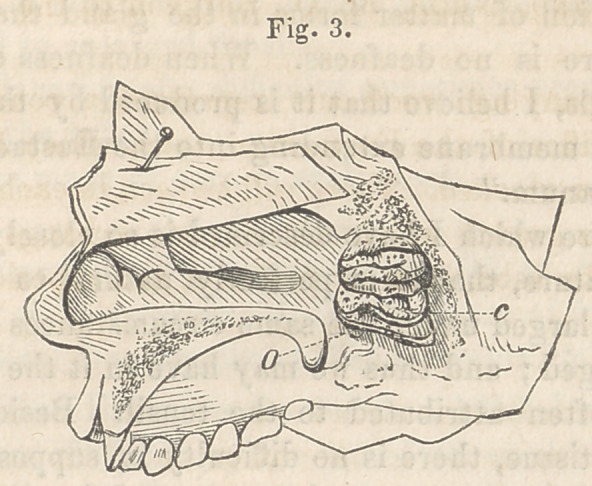# On Obstruction of the Pharyngeal Orifice of the Eustachian Tube

**Published:** 1853-10

**Authors:** Jno. Neill

**Affiliations:** Surgeon to the Pennsylvania Hospital


*On Obstruction of the Pharyngeal Orifice of the Eustachian Tube.* By JNO. NEILL, M. D., Surgeon to the Pennsylvania Hospital.

The accompanying wood cuts represent a condition of the pharyngeal orifice of the Eustachian tube, which I believe has hitherto been overlooked. There will be seen in each of the specimens figured, a growth which completely overhangs and occludes the orifice of the tube, from its posterior and superior border.

Fig. 1.

Fig. 1, a. The growth seems to consist of a thickened fold of mucous membrane, in which the mucous follicles are very distinct.

Fig. 2.

Fig. 2, b. The structure resembles more in appearance and in size the tonsil, but when carefully examined, is found to be composed of several folds of mucous membrane, thickly studded with mucous glands.

Fig. 3.

Fig. 3, c. This mass also resembles the tonsil in structure, presenting those wide-mouthed follicles which are characteristic of the gland.

The subjects from which these specimens were obtained were not known to be affected with these peculiarities previous to death, nor were they selected for the purpose of illustrating this condition; and, therefore, it is either a remarkable coincidence that this pathological formation should be found in three instances under such circumstances, or it is to be inferred that the condition is a very common one.

I am inclined to think that this condition of the orifice of the tube exists quite frequently, especially in children, and that it is a common source of temporary deafness.

How many cases of deafness in children are considered to be dependent upon enlarged tonsils, and yet how few are the instances in which the tonsil is sufficiently enlarged to encroach upon the orifice of the tube? In fact, the distance between the tonsil and the orifice is so great as to induce some to believe that enlarged tonsils are never the cause of deafness; and yet we certainly do find this enlargement coexistent with deafness, though it may not be the cause of it. Mr. Wilde says, "I have never seen a preparation showing the greatest possible degree of enlargement of the tonsil, in which it pressed upon the trumpet-mouth of the Eustachian tube. Anatomists will, therefore, find it as difficult to believe that enlarged tonsils produce deafness, as practical surgeons to believe that their removal can in any way relieve the loss of hearing. Even in cases of cynanche tonsillaris, when so large a collection of matter forms in the gland that suffocation threatens, there is no deafness. When deafness co-exists with enlarged tonsils, I believe that it is produced by the thickening of the mucous membrane extending into the Eustachian tube or into the tympanum."

The structure which I have described is so closely allied to the tonsil in its nature, that it is perfectly natural to suppose that it would be enlarged under the same circumstances by which the tonsil is enlarged; and thus we may have in it the real cause of deafness so often attributed to the tonsil. Besides, from the nature of the tissue, there is no difficulty in supposing that this hypertrophy of the mucous membrane around the orifice may exist, independently of any enlargement of the tonsil. That this structure is a hypertrophy of the mucous membrane is evident from its existing on both sides in each case, whereas, were it a polypus or an epithelial formation, it is hardly probable that it would occur on both sides at the same time.

It is somewhat remarkable that such a structure has not been noticed in the frequent catheterizations of the tube. In two of these specimens the orifice (*o*) was so concealed that a probe could not be introduced from the pharynx even, without elevating the overhanging mass, and it would have been next to impossible to have introduced a catheter in the ordinary manner to determine the permeability of the tube.

The usual mode also of determining the permeability of the tube, by closing the nostril and mouth and making forcible expiration must fail, when such a condition of the orifice exists, although the upper part of the tube be entirely free from constriction. Forcible expiration, would, in fact, force this growth more tightly into the orifice, as a cork into the mouth of a bottle.

The position of this morbid structure is such that it cannot be reached by the knife, nor could caustic be applied very satisfactorily through the nose. The only manner of reaching the difficulty would be by a properly curved caustic holder introduced through the mouth.

Inspection of these specimens may justify the following reflections.

1. That there is a pathological condition of the orifice of the Eustachian tube hitherto not described.

2. A form of deafness may depend on its existence, which has been ascribed to enlarged tonsils.

3. An explanation of a difficulty sometimes preventing catheterization of the tube.

4. The true mode of cure in such cases.

## Figures and Tables

**Fig. 1. f1:**
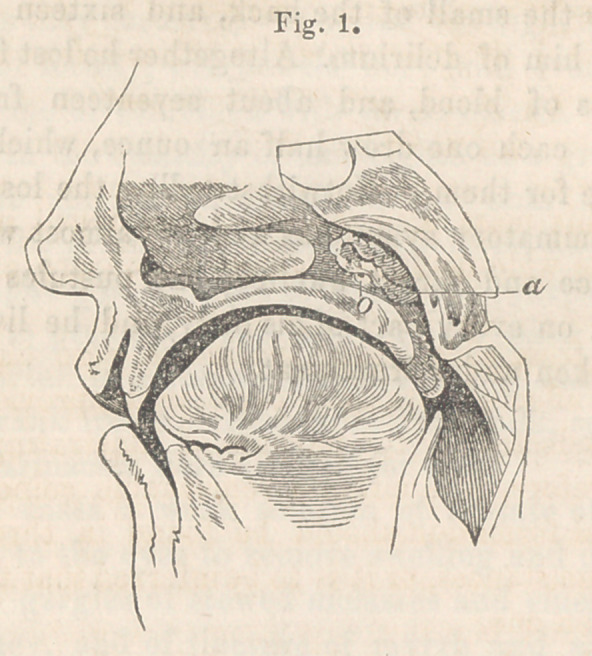


**Fig. 2. f2:**
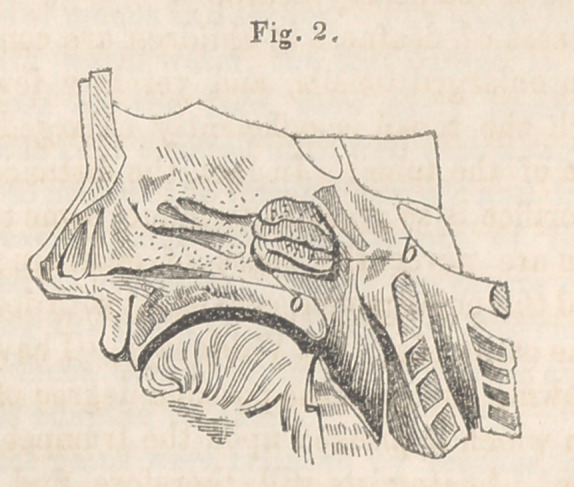


**Fig. 3. f3:**